# OSeac: An Online Survival Analysis Tool for Esophageal Adenocarcinoma

**DOI:** 10.3389/fonc.2020.00315

**Published:** 2020-03-06

**Authors:** Qiang Wang, Zhongyi Yan, Linna Ge, Ning Li, Mengsi Yang, Xiaoxiao Sun, Longxiang Xie, Guosen Zhang, Wan Zhu, Yunlong Wang, Yongqiang Li, Xianzhe Li, Xiangqian Guo

**Affiliations:** ^1^Cell Signal Transduction Laboratory, Bioinformatics Department of Predictive Medicine, Bioinformatics Center, Henan Provincial Engineering Center for Tumor Molecular Medicine, School of Software, School of Basic Medical Sciences, Institute of Biomedical Informatics, Henan University, Kaifeng, China; ^2^Department of Anesthesia, Stanford University, Stanford, CA, United States; ^3^Henan Bioengineering Research Center, Zhengzhou, China; ^4^Department of Thoracic Surgery, The Affiliated Nanshi Hospital of Henan University, Nanyang, China

**Keywords:** EAC, prognostic, survival analysis, biomarker, web server

## Abstract

Esophageal Adenocarcinoma (EAC) is one of the most common gastrointestinal tumors in the world. However, molecular prognostic systems are still lacking for EAC. Hence, we developed an **O**nline consensus **S**urvival analysis web server for **E**sophageal **A**deno**c**arcinoma (OSeac), to centralize published gene expression data and clinical follow up data of EAC patients from The Cancer Genome Atlas (TCGA) and Gene Expression Omnibus (GEO). OSeac includes 198 EAC cases with gene expression profiling and relevant clinical long-term follow-up data, and employs the Kaplan Meier (KM) survival plot with hazard ratio (HR) and log rank test to estimate the prognostic potency of genes of interests for EAC patients. Moreover, we have determined the reliability of OSeac by using previously reported prognostic biomarkers such as *DKK3, CTO1*, and *TXNIP*. OSeac is free and publicly accessible at http://bioinfo.henu.edu.cn/EAC/EACList.jsp.

## Introduction

Esophageal cancer is a common malignant tumor of digestive tract. The incidence of esophageal cancer is eighth in all tumors and sixth in fatal cancer (1). EAC is one of the most common histological types of esophageal cancer (2, 3) and has increased markedly in Western countries in recent decades (4, 5). Esophagectomy with the addition of perioperative chemotherapy or chemoradiotherapy had improved prognosis of EAC (6–8), however not all EAC patients got cured. The use of molecularly targeted agents are not satisfied so far for EAC and it has lagged behind other cancers (9). Therefore, it is necessary to identify new predictive and prognostic biomarkers for EAC patients to improve clinical outcome.

The expression of genes has been shown to guide the prognosis of cancer patients (10). Three genes *DKK3, TXNIP*, and *CTO1* have been reported as prognosis biomarkers in EAC patients (11–13). However, these biomarkers need independent further validation to increase their sensitivity and specificity before clinical application. The advanced bioinformatic methods and resources have been developed for breast cancer, bladder cancer, esophageal squamous cell carcinoma, leiomyosarcoma, and lung cancer to analyze the prognostic abilities of genes (14–19), and greatly facilitate the development of cancer prognostic biomarkers. However, there is a lack of prognostic analysis system for EAC.

In this study, an online prognostic analysis tool for EAC was developed. It can not only rapidly evaluate the value of prognostic molecular biomarkers, but also provide the opportunities to identify the potential new therapeutic targets for EAC patients.

## Materials and Methods

### Data Collection

Three EAC datasets were collected from GEO (http://www.ncbi.nlm.nih.gov/geo/) and TCGA (http://cancergenome.nih.gov), these datasets include gene expression profiles and clinical follow-up information of EAC (Table 1).

**Table 1:** Datasets used in OSeac.

**Dataset**	**Platform**	**Stage** **(%I/II/III/IV/NA)**	**References**	**No. of samples**
TCGA	RNAseq	11.25/27.5/33.75/ 6.25/21.25	(20)	80
GSE13898	GPL6102	15.71/34.29/10.00/ 0.00/40.00	(21)	70
GSE19417	GPL4372	–	(22)	48

### Development of OSeac

The OSeac server is developed as we previously described (17–19), and hosted in a windows server and adopts Appache Tomcat as web application server. Use HTML and JSP for the front end page and server side code is compiled to Java. The R package “Rserve” as a middleware enables Java to call programs written in R language. The SQL Server database is used as backend database which stores data of the gene expression profiles and clinical information. The central server for OSeac can be accessed at http://bioinfo.henu.edu.cn/EAC/EACList.jsp.

## Results

### OSeac Establishment and Usage

We collected gene expression profiles and clinical follow-up information of 198 EAC patients from TCGA and GEO databases, and established OSeac to measure the association between a queried gene and patient outcome by Kaplan Meier plot. Before performing analysis, users can set up filter conditions by confounding clinical factors such as stage and gender. Four outcome terms including overall survival (OS), disease specific survival (DSS), disease-free interval (DFI) (23), and progression-free interval (PFI) (23) can be determined in OSeac.

OSeac provides the main functions that evaluate the prognostic value of gene of interests. When users input official gene symbol in the textbox, select an individual dataset or combined datasets [the combined datasets mean that all the patients from individual datasets were combined as a pool after they were stratified into subgroups (high vs. low) based on the expression level of inputted gene in each dataset], then choose an appropriate cutoff value for gene expression (including median, quartile or 30%) and click the “Kaplan-Meier plot” button, the server will generate survival curves and display HR with 95% confidence intervals and *P*-value on the output web page. Currently, five clinical factors were set as optional factors to limit the analysis in a subgroup of EAC patients for special needs from different researchers, these clinical factors include TNM stage, gender, race, grade and treatment response.

### Validation of Previously Published EAC Biomarkers in OSeac

**Figure 1:**
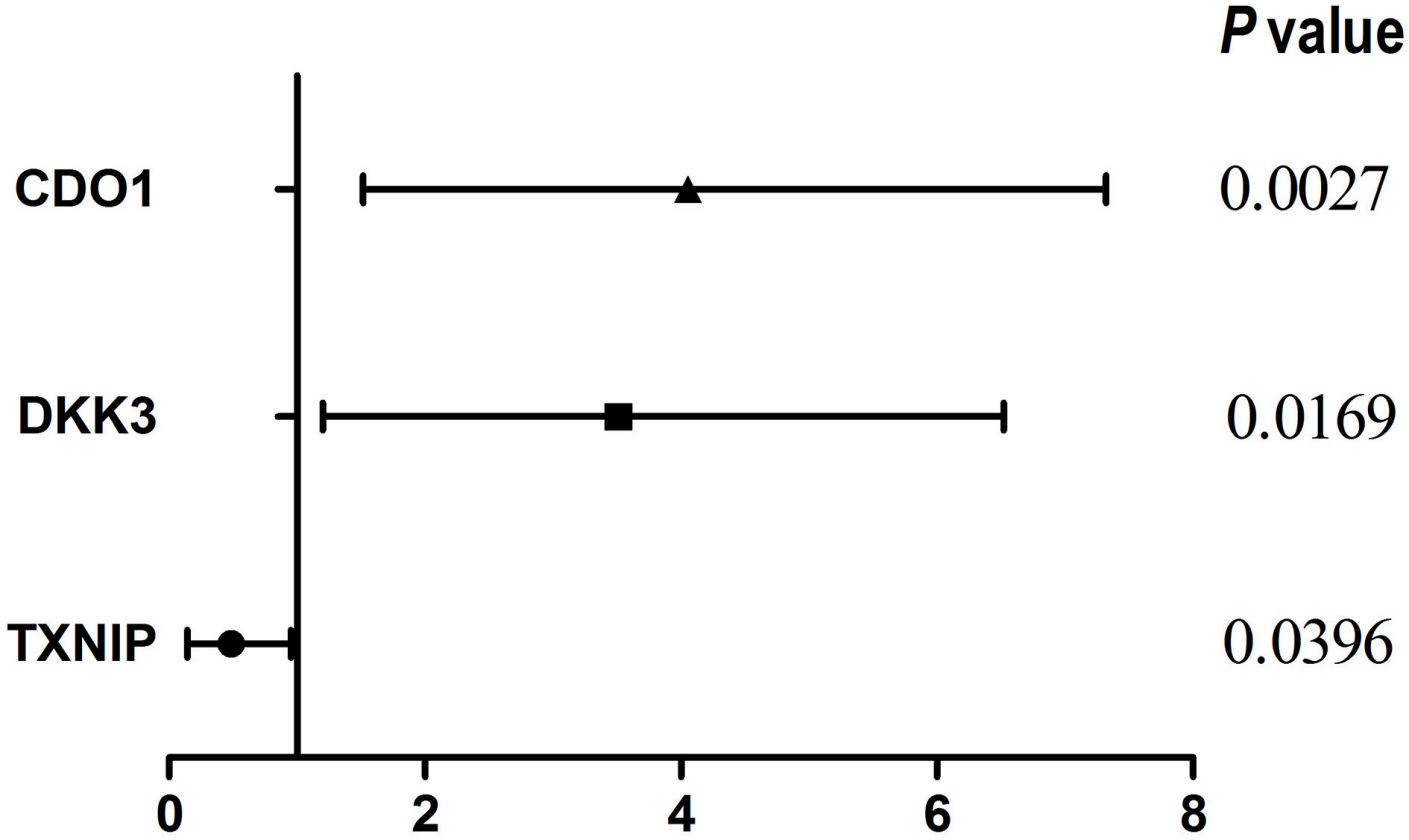
Forest plot of three known prognosis biomarkers.

We evaluated three published prognostic biomarkers, including *DKK3, CDO1*, and *TXNIP* (Table 2, Figure 1) in OSeac. As shown in Table 2 and reported in original literatures, the higher expression of gene *TXNIP* in EAC patients implies a significant better overall survival rate, whereas higher expression of other two genes predict a significant worse overall survival. This result shows the validity and reliability of OSeac in determining the prognostic potency of genes of interests.

**Table 2:** Performance of previously published prognostic biomarker in OSeac.

**Gene symbol**	**Literature data**				**OSeac data**			
	***n***	***P*-value**	**Prognostic value**	**References**	**HR (95% CI)**	***P*-value**	**Survival**	**Validation results**
*DKK3*	116	<0.0500	Poor	(11)	2.8012 (1.2033–6.5212)	0.0169	OS	√
*TXNIP*	228	0.0020	Good	(12)	0.3698 (0.1424–0.9538)	0.0396	OS	√
*CDO1*	38	0.0360	Poor	(13)	3.3329 (1.5175–7.3202)	0.0027	OS	√

*OS, overall survival; HR, hazard ratio; CI, confidence interval; DKK3, dickkopf WNT signaling pathway inhibitor 3; CDO1, cysteine dioxygenase type 1; TXNIP, thioredoxin interacting protein*.

### Discovering Potential Biomarkers for EAC in OSeac

**Figure 2:**
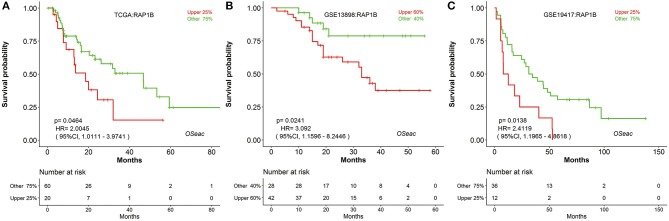
Kaplan Meier plots of a potential prognosis biomarker *RAP1B* in TCGA **(A)** GSE13898 **(B)** and GSE19417 **(C)** “upper 25%” and “other 75%”: sub-categorizing approach. After the expression level of inputted gene is sorted, take the patients with top 25% high expression level as “upper 25%” subgroup and remaining patients as “other 75%” subgroup.

In addition, to further test the prediction power of OSeac, we choose a known prognostic marker of gastric cancer (GC) (24) to analyze it in OSeac. We examined prognostic potency of *RAP1B* for EAC patients by OSeac, and found that *RAP1B* was significantly associated with unfavorable OS in TCGA (*P* = 0.0464, HR: 2.0045, 95% CI: 1.0111–3.9741), GSE13898 (*P* = 0.0241, HR: 3.0920, 95% CI: 1.1596–8.2446) and GSE19417 (*P* = 0.0138, HR: 2.4119, 95% CI: 1.1965–4.8618). This suggests that *RAP1B* could be a potential prognostic indicator of poor OS for EAC (Figure 2).

## Discussion

In this study, we have developed an online tool OSeac to analyze prognostic biomarkers in EAC. As most researchers may concern the accuracy rate and potential error from OSeac, for example, we performed the prognosis analysis for one gene each time in OSeac, the gene will be regarded significant for prognosis when *P*-value is < 0.05, while there are more than 20,000 genes in human genome, which means we may get 1,000 significant prognostic genes by chance when we do genome-wide repeated measurements. In addition, the EAC expression datasets may come from different analyzing platforms/technologies, or from different ethnicities, all these may influence the results of prognosis analysis. Nevertheless, in order to increase the specificity of prognosis biomarkers, we did our best to collect EAC expression datasets from GEO, TCGA and literatures as many as possible, and offered the opportunities to do independent validation across different EAC cohorts, which as we know is most important for biomarker development. Finally, to determine the performance of OSeac, three published EAC prognostic biomarkers were assessed in OSeac, and all of them reach statistical significance for prognosis in OSeac as expected, indicating the good performance of OSeac in prognostic biomarker screening. OSeac could also be used to screen novel prognosis biomarker for EAC, for example, *RAP1B* contributes to tumor malignant behavior and poor prognosis in GC (24). Using OSeac to assess the prognostic value of *RAP1B* in EAC, we found that *RAP1B* is a potential unfavorable prognostic indicator for EAC patients.

In conclusion, OSeac could help clinicians, biologists, and researchers to easily evaluate prognostic significance of genes of interests in EAC. The limitation of OSeac is that currently OSeac contains only 3 datasets and 198 samples, the sample number is relatively low, therefore, we will keep update and expand OSeac when new EAC datasets are available.

## Data Availability Statement

Publicly available datasets were analyzed in this study. This data can be found here: http://bioinfo.henu.edu.cn/EAC/EACList.jsp.

## Ethics Statement

Written informed consent was obtained from the individual(s), and minor(s)' legal guardian/next of kin, for the publication of any potentially identifiable images or data included in this article.

## Author Contributions

XG: study concept and design. ZY, LX, XG, LG, NL, WZ, YW, and XL: acquisition of data. QW, LX, XG, MY, XS, WZ, YW, and XL: analysis and interpretation of data. QW, LX, GZ, YL, XG, and XL: draft of the manuscript. QW, ZY, GZ, LX, WZ, and XG: critical revision of the manuscript for intellectual content.

### Conflict of Interest

The authors declare that the research was conducted in the absence of any commercial or financial relationships that could be construed as a potential conflict of interest.
